# Stereotypes Possess Heterogeneous Directionality: A Theoretical and Empirical Exploration of Stereotype Structure and Content

**DOI:** 10.1371/journal.pone.0122292

**Published:** 2015-03-26

**Authors:** William T. L. Cox, Patricia G. Devine

**Affiliations:** Department of Psychology, University of Wisconsin—Madison, Madison, Wisconsin, United States of America; University College London, UNITED KINGDOM

## Abstract

We advance a theory-driven approach to stereotype structure, informed by connectionist theories of cognition. Whereas traditional models define or tacitly assume that stereotypes possess inherently Group → Attribute activation directionality (e.g., *Black* activates *criminal*), our model predicts *heterogeneous stereotype directionality*. Alongside the classically studied Group → Attribute stereotypes, some stereotypes should be bidirectional (i.e., Group ⇄ Attribute) and others should have Attribute → Group unidirectionality (e.g., *fashionable* activates *gay*). We tested this prediction in several large-scale studies with human participants (*N_Combined_* = 4,817), assessing stereotypic inferences among various groups and attributes. Supporting predictions, we found heterogeneous directionality both among the stereotype links related to a given social group and also between the links of different social groups. These efforts yield rich datasets that map the networks of stereotype links related to several social groups. We make these datasets publicly available, enabling other researchers to explore a number of questions related to stereotypes and stereotyping. Stereotype directionality is an understudied feature of stereotypes and stereotyping with widespread implications for the development, measurement, maintenance, expression, and change of stereotypes, stereotyping, prejudice, and discrimination.

## Introduction

Research on stereotypes and stereotyping explores what comes to mind when a given social concept is activated (e.g., *Black* brings to mind *criminal*), and when, how, and why one social concept (e.g., *Black*) brings to mind another (e.g., *criminal*). Understanding the content, process, and structure of cognitions related to social groups is important because these cognitions form the building blocks of prejudice, discrimination, and oppression [1–6]. The interpretation of every finding in the stereotyping and prejudice literatures depends at least in part upon the researcher’s underlying theoretical model of stereotypes and stereotyping. Different models make different assumptions about, for instance, how stereotypes are structured in memory, how they are activated, and what is involved in changing them [7]. Clear scientific progress toward understanding stereotyping and prejudice, therefore, requires a clear understanding of the cognitive architecture that underlies stereotypes. As Hilton and von Hippel lamented, however, many researchers’ models and definitions of stereotypes and stereotyping are imprecise—or worse, unspecified—resulting in considerable ambiguity about the nature of stereotypes and stereotyping [7].

Because stereotypes are cognitive knowledge structures, many stereotype models have built on developments in cognitive psychology (see [7–8] for reviews). Social psychologists have advanced models that conceptualize stereotypes as, for instance, prototypes, collections of exemplars, or organizational schemas, each approach paralleling similar models of cognition put forth by cognitive psychologists [7]. Within the last 30 years, connectionist theories of cognition, which are rooted in and constrained by assumptions about how the brain works, have risen to prominence in cognitive psychology [9–13]. Though there are a few exceptions (e.g., [14–18]), however, connectionism has gained little traction in social psychology generally or in the study of stereotypes more specifically [9–10]. In cognitive psychology, connectionist approaches are compelling to many because their conceptualization of cognition is closely tied to brain biology and because they have provided a number of fruitful insights and advances in the cognitive literature [11–13]. We contend that connectionism can be similarly useful to the study of stereotypes.

In the present article, we use connectionism as a theoretical foundation to develop a framework for understanding stereotypes and stereotyping. This framework leads us to some specific predictions about stereotype structure that do not follow as readily from traditional models. To test these predictions, we explore and describe the structure of a variety of stereotypes related to different social groups (e.g., *Black men*, *gay men*) in several large-scale studies with human participants. We employ traditional methods for assessing stereotype content and structure, but use them in a new way that reveals features of stereotype structure that have largely been unexplored in prior work. These efforts yield a set of data matrices that map the groups’ stereotype networks, which are valuable resources that can be used by other researchers to explore a number of questions related to stereotypes and stereotyping. The present work provides a rich theoretical and empirical foundation from which to generate novel predictions and insights concerning core issues in the stereotype literature, including stereotype formation, activation, measurement, and change, and how stereotypes relate to prejudice and discrimination.

### A Connectionist-Inspired Approach to Stereotype Structure

Although connectionism is often perceived as a set of methods (e.g., computational modeling), it is more precisely a theoretical framework for how knowledge is learned, represented, and retrieved [9–13, 19]. The central assumption of connectionist approaches is that cognition arises from brain activity and thus should operate in a way that reflects the brain’s mechanics. Brains are composed of vast networks of connected neurons, and it is the patterns of activation across these neural connections that store knowledge and give rise to cognitive processes. Neurons in the brain display fractal geometric properties: the features that characterize neural patterns at one scale (e.g., the relationship between two individual neurons) can also be seen in neural patterns at other scales (e.g., the relationship between two clusters of neurons) [20, 21]. Because of this fractal scaling, the basic mechanics of neurons and synapses are theorized to scale upwards to cognitive knowledge structures. In other words, knowledge structures should behave in ways that reflect the mechanical principles of their constituent neural strata. The core implication of this approach is that any theory of cognition should both *reflect* the principles of basic neural anatomy and be *constrained by* those principles, with cognitions represented as and behaving like distributed networks of undifferentiated, neuron-like units (for more comprehensive review, see [9–13, 19]).

From this perspective, then, what is a stereotype, and how would it differ from traditional conceptualizations? Traditional models conceptualize the problem of stereotyping as linking attributes (e.g., traits, characteristics) with social groups in memory. According to this approach, then, a stereotype is a composite cognitive representation of a social group category (e.g., *Black men*) and the attributes (e.g., *criminal*, *poor*, *athletic*) commonly associated with members of that category [8]. When the social group category is activated, activation of the attributes follows and it is this pattern of activation that is considered to be stereotyping. In such models, the social group takes on prominence and the attributes are subordinated in that they become relevant in so far as the social group category is activated.

Connectionist frameworks, in contrast, largely discard the notion of different types of cognitive units, especially the notion of categories [12]. What are referred to as “groups” versus “attributes” in traditional models, we argue, are distinguished by their social importance and the interests of the researcher. Groups and attributes are social concepts and do not reflect distinct types of cognitive units [2]. Groups, attributes, and their relationships are all stored as connection weights among undifferentiated units. As such, the extent to which a concept label refers to a socially important group is only one feature that may characterize or influence cognitive structure; it is not a feature that makes social group concepts a special type of cognitive unit. In the present work, therefore, we use the term “group” merely to specify the focal group of interest, and the term “attribute” refers to any and all other concepts (e.g., traits, behaviors, features, roles) that may be linked to that group of interest.

Removing what, from our perspective, is an artificial cognitive unit distinction sets our model apart from both classic models [22–24] and some modern models [14, 25, 26] that give special status to “groups.” This seemingly small adjustment has major implications for a number of key topics [2], and it yields our definition of a stereotype. A *stereotype* is a learned association, or link, between two social concepts (e.g., *Black*, *criminal*) that are not defining features for one another (for greater detail, see [2]).

Note that placing “groups” and “attributes” on equal footing requires defining a stereotype as an association between only two concepts (e.g., the *Black*–*criminal* stereotype is treated as potentially distinct from the *Black*–*poor* stereotype). To assess stereotype structure, then, we must independently assess each component stereotype link associated with our group of interest. At first glance, this seems an easy feat—since the earliest stereotyping work, researchers have assessed the probability that certain attributes are brought to mind by a given social group concept [22]. Treating groups and attributes as the same type of cognitive unit, however, requires an adjustment to this approach. We must assess not only the probability that the group brings to mind the attribute (Group → Attribute), as in traditional work, but also the probability that the attribute brings to mind the group (Attribute → Group). In some cases, these probabilities may be equivalent (e.g., A elicits B just as often as B elicits A), yielding a bidirectional stereotype (A ⇄ B). In other cases, however, these probabilities may differ, yielding a stereotype that is more unidirectional, favoring in one direction (A → B) or the other (B → A). These different possibilities illustrate *heterogeneous stereotype directionality*. We argue that directionality is an understudied dimension of stereotype structure that is fundamental to understanding stereotypes and stereotyping.

**Fig 1:**
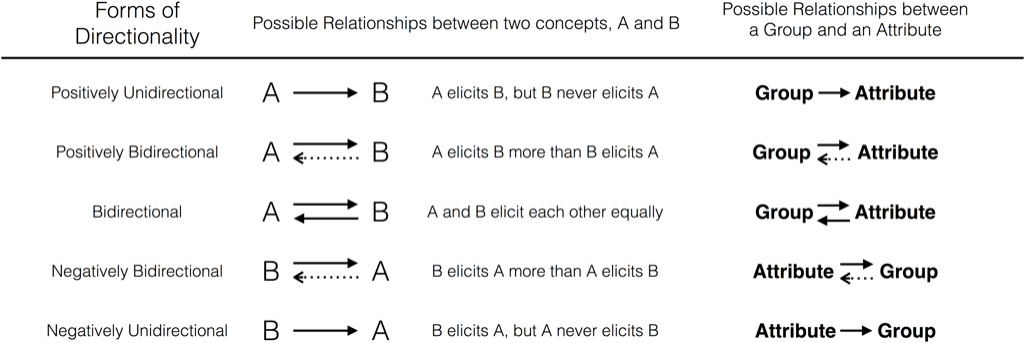
Possible Forms of Directionality.

Formalizing the reasoning above, the structure of any Group—Attribute stereotype must have directionality that falls somewhere on a spectrum from positive unidirectionality (Group → Attribute) to bidirectionality (Group ⇄ Attribute or Attribute ⇄ Group) to negative unidirectionality (Attribute → Group), as displayed in Fig. 1. This formulation of heterogeneous stereotype directionality diverges from classic perspectives, which have largely emphasized stereotyping as a unidirectional process that relies upon *a priori* activation of a social category (e.g., [23–24]). This focus on positive, Group → Attribute unidirectionality makes sense practically, because stereotyping and prejudice research is most often concerned with the negative consequences of membership in an oppressed group. In support of our notion of heterogeneous stereotype directionality, however, recent work suggests that patterns of stereotype activation are more complex than the Group → Attribute models typically assume. For example, some recent work [27, 28] has demonstrated that, when the defining features of group membership are not visible, perceivers rely on visible stereotypic attributes to draw conclusions about group membership (e.g., inferring that a fashionable man is gay)—thus showcasing stereotyping in the Attribute → Group direction.

Other work has provided suggestive evidence that certain stereotypes may be bidirectional [29–30] or have negative, Attribute → Group directionality [27–28, 31–33]. We use the term “suggestive” because this body of work has demonstrated that stereotypes *can* be activated or used in the Group → Attribute and Attribute → Group directions, but it has not directly assessed the extent to which activations are *more likely* in one direction versus the other. As such, this work is consistent with, but does not test, the possibility that stereotypes have heterogeneous directionality. Because no prior work has *compared* Group → Attribute and Attribute → Group activations, these past patterns are also consistent with the alternative that all stereotypes are inherently bidirectional—in which case evidence of apparent unidirectionality arises only as an artifact of unidirectional experimental tasks. We, however, find the notion of heterogeneous stereotype directionality more compelling, because it is more consistent with basic learning processes, real-world observations, and even neural mechanics.

Indeed, our formulation of heterogeneous directionality directly reflects neural mechanics [34] and thus exhibits internal consistency with our connectionist approach. Because individual synapses are inherently unidirectional from axon to dendrite, the net connection between any two neurons must fall on a directionality spectrum like that of Fig. 1. These mechanics operate at the deeper, neural level of analysis, and, through the lens of connectionism, we expect to see these principles reflected at higher levels of analysis. Accordingly, directionality appears as a crucial consideration across many domains, from basic learning in nonhuman animals through abstract logical reasoning (see Table 1), and we see no reason for stereotypes to be an exception.

**Table 1:** Examples of Directionality Across Domains and Levels of Analysis.

Level of Analysis/Domain	Example
Logical Reasoning	Fallacy of the converse; affirming the consequent: If all dogs are mammals, that does not imply that all mammals are dogs
Similarity Judgments	Asymmetry of Similarity Judgments: North Korea is rated as more similar to China than China is to North Korea [35]
Causal Reasoning	Reasoning from cause to effect (predictive reasoning) has different properties than reasoning from effect to cause (diagnostic reasoning). [36–38]
Cultural Knowledge	In cultures familiar with “Knock-Knock” jokes, the well-known verbal prompt, “Knock Knock” will almost invariably elicit the response “Who’s There?” from even a total stranger. If, however, one were to reverse the direction and ask someone “Who’s There?”, it is unlikely that “Knock Knock” would even occur to anyone as a response.
Semantic/Lexical	The statistical structure of language is directional. For example, Noun-Noun Pairs (e.g., SKI JACKET) lose their meaning when reversed (e.g., JACKET SKI) [39–40].
Basic Association Learning; Learning in Nonhuman Animals	Learning tasks with nonhuman animals provide little or no evidence of emergent symmetry; when an organism learns a relationship A → B, it does not spontaneously learn the opposite, B → A. This tends to be the case with humans as well. Learning that a red light is followed by a shock will not lead one to expect a red light following a shock (e.g., [41]).
Cortical Signal Flow	Cortical signal flow is reversed in visual imagery versus visual perception. [42]
Neural Mechanisms	Neurons send signals with axons and receive them with dendrites [34, 43].

Examples of directionality as a key consideration across many domains and levels of analysis. Directionality refers to the notion that the relationship from A to B (A → B) is independent from the relationship from B to A (B → A), and our formulation of heterogeneous directionality directly reflects neural mechanics [34]. Individual synapses between neurons are inherently unidirectional, from one neuron’s axon to the other’s dendrite. Therefore, the connection between any two neurons (or, scaling up, clusters of neurons), A and B, must have a relationship that falls on a directionality spectrum, from positively unidirectional (A’s axons connect to B’s dendrites; A → B) to bidirectional (A’s and B’s axons connect to each other’s dendrites; A ⇄ B) to negatively unidirectional (B’s axons connect to A’s dendrites; B → A).

Because observed cognitive structure emerges from the statistical structure of human learning experiences [12, 34, 44–45], understanding the directionality of a particular stereotype, and regularities or irregularities in the directionality of different stereotypes, should give us insights into how the stereotypes are learned, reinforced, or used. For instance, if a stereotype possesses negative directionality (e.g., *fashionable* → *gay*), this may indicate that it functions primarily as a social categorization tool [28]. Or, if a stereotype is bidirectional, this may indicate that the two linked concepts tend to be presented simultaneously, or perhaps that associations in each direction developed somewhat independently. For these reasons, we argue that exploring directionality has the potential to generate a wide range of insights about stereotypes and stereotyping processes.

### The Paradigm

For a given Group—Attribute stereotype, we must *independently* assess both the probability that the group brings to mind the attribute (Group → Attribute) and the probability that the attribute brings to mind the group (Attribute → Group). This requirement precludes the use of any experimental tasks that present both concepts as stimuli to a given participant (e.g., attribute checklists, sequential priming tasks), because such tasks activate associations in both directions, leaving us unable to distinguish activations in one direction versus the other. To circumvent this problem, we use a between-subjects design in which people are randomly assigned to respond to a *single* stimulus, either a stereotyped group (e.g., *Black*) or a stereotypic attribute (e.g., *criminal*) drawn from a larger set of concepts thought to make up a given stereotype network. Participants report the associates brought to mind by the single stimulus, allowing us to establish unidirectional association norms for each stimulus concept [3, 46–49]. Using a pure production task and a completely between-subjects experimental design ensures that we are assessing unidirectional relationships within each condition.

The unit of analysis in the present work is the stereotype link, and each link’s directionality is assessed via a two-condition, between-subjects experiment. To test the *Black—athletic* link, for example, participants are randomly assigned to either see the stimulus phrase “A man who is Black,” or “A man who is athletic.” The dependent variable is whether participants respond with the concept they were *not* given as a stimulus (e.g., whether *Black* brought to mind *athletic* or *athletic* brought to mind *Black*). This design allows us to quantify the extent to which a given stereotype link (e.g., *Black*–*athletic*) is bidirectional (*Black* ⇄ *athletic*) or unidirectional in either direction (*Black* → *athletic* or *athletic* → *Black*).

### The Present Work

The primary goal of the present work is to assess the extent to which stereotypes possess heterogeneous directionality by documenting and describing the directional structure of various stereotypes and stereotype networks of interest. To that end, in Study 1 we mapped the structure of the stereotype networks related to two stigmatized groups historically of interest to social psychologists. Studies 2 and 3 provided methodological validations of Study 1 and tested some alternate explanations for its findings. Specifically, Study 2 examined whether social desirability concerns may have unduly influenced Study 1’s patterns, and Study 3 evaluated whether Study 1 may have improperly capitalized on random chance. Lastly, Study 4 trades Study 1’s depth within two groups’ stereotype networks for breadth across multiple groups, mapping the structure of a wide selection of other stereotypes.

A secondary goal of the present work was to use our data to create a publicly available resource for other researchers interested in exploring stereotype structure. The data matrices arising from the present work map the structure of each stereotype network, quantifying the directional structure of the individual relationships between the group concept of interest (e.g., *Black men*) and each of its various stereotypic attributes (e.g., the *Black*–*criminal* link, the *Black*–*poor* link), as well as the relationships, if any, among the various attributes themselves (e.g., the *criminal*–*poor* link). For each of our studies, we provide both the raw participant data and the coded data matrices, which can be useful, flexible resources for future work, to directly test hypothesis within the data sets, or merely as reference tools for generating novel research questions and hypotheses.

## Study 1

Study 1 explored stereotypes related to Black men and gay men. We chose these two groups in particular for multiple reasons. Stereotypes about these groups have received extensive attention by stereotyping and prejudice researchers, which 1) allows us to draw upon the theoretical and empirical foundations of past work, and 2) makes it more likely that the present exploration can be useful to future work with these groups. Also, based on past work and our own theorizing, stereotypes related to these two groups seem especially likely to reveal heterogeneous directionality. Because the defining features of gay male group membership (i.e., same-sex attraction, gay identity) are not visible, people often rely on stereotypic cues (e.g., fashion) to infer that men are gay [27–28, 50–51]. Gay male stereotypes, therefore, are often used in the negative, Attribute → Group direction, which suggests to us that they may be more likely to possess negative directionality. Because race is a visible, salient group status, people probably do not often rely upon stereotypic cues to activate the concept *Black*, except perhaps in situations when race is not as obvious (e.g., over the phone [52], cf. [25, 32]). Black male stereotypes may therefore be likely to possess either positive directionality (Group → Attribute) or bidirectionality. We anticipate, therefore, that stereotypes related to these two groups are likely to reveal heterogeneous directionality, making them an ideal starting point for the present work. We later extend our exploration of directionality to stereotypes related to many other social groups in Study 4.

### Method

#### Design and participants

We examined many different attributes stereotypically associated with gay men and Black men, to provide as complete a picture as possible for these two groups’ stereotype networks. Based on our anticipated sample size, we decided that Study 1 would have 60 conditions total, with the groups and their various stereotypic attributes each being a single between-subjects condition. In all, 2295 undergraduates participated. Before coding any of the data, we excluded participants who reported being in the U.S. for fewer than 4 years (to insure familiarity with U.S. stereotypes; *n* = 190) or who provided fewer than four of the five responses our task requested (i.e., less than 80% completion of the task/missing more than a single data point, *n* = 41). The responses from these excluded participants were never coded. Lastly, one condition (*n* = 15) was included as part of another, unrelated project. After these exclusions, therefore, each of 2049 undergraduates was randomly assigned to one of 59 conditions, yielding a sufficient per-condition sample size (*M*_*n*_ = 34.7, *sd*_*n*_ = 2.56). All the experiments in this article were approved by the Social and Behavioral Sciences Institutional Review Board at the University of Wisconsin—Madison. All materials and data from this article are available publicly at www.sciencecox.com/pub/ds15.

#### Stimulus set

We generated an extensive list of stereotypic traits, behaviors, professions, and interests associated with Black men and gay men. We retrieved many from the past literature [3, 22, 28, 53–54], which is biased toward examining stereotypes with positive directionality, because the past literature relies on methods that provide a group label and ask for stereotypic attributes. Because of this bias, we also culled stereotypic traits from the media, literature, and personal experiences, and we elicited stereotypes of different directionality from undergraduates using discussion and free response questions (e.g., “How would you be able to tell if a man was gay without being able to ask him?”). Using these sources yielded a list of over 1200 candidate phrases that could be used as stimuli in the study.

A coder who was blind to the hypotheses of the study sorted the extensive list into categories (e.g., “steals things,” “is a criminal,” “kills people,” and “breaks into people’s houses” were grouped into the category *criminal*). The coder then identified the item that was either the most prototypical of each category (e.g., “is a criminal” from the *criminal* category) or was a prominent exemplar of it (e.g., “is a Cher fan” from the *musical artist fan* category). Based on the projected size of our sample, we asked this hypothesis-blind coder to select a total of 57 stereotypic attributes.

The concepts were all placed in phrases with the stem “A man who ______”, which bounded the task to cognitions about a male person (e.g., “A man who is Black.” “A man who is a criminal.”). Our full set of stimulus items included 20 stereotypic attributes related to *Black men* (e.g., “A man who is a criminal”), 20 stereotypic attributes related to *gay men* (e.g., “A man who is fashionable”), 17 stereotypic attributes that could apply to both groups (e.g., “A man who is overly sexual”), and 2 phrases for the groups of interest (“A man who is Black” and “A man who is gay”). These stimulus phrases are displayed in Table 2, and include an assortment of stereotypic concepts, including professions (e.g., *rapper*, *nurse*), physical attributes (e.g., *tall*, *well groomed*), personality characteristics (e.g., *friendly*, *dramatic*), clothing (e.g., *baggy clothing*, *tight clothing*), and interests/activities/preferences (e.g., *plays basketball*, *likes shopping*), among others.

**Table 2:** Stimulus Phrases for Black and Gay Stereotype Networks in Study 1.

**A man who…**	is Black	is overly sexual	is gay
	has poor articulation of words	uses lots of hand gestures when he talks	has a lisp
	is strong	is well dressed	is well groomed
	likes hip hop music	likes Beyoncé	is a Cher fan
	wears baggy clothing	wears tank tops	wears tight clothing
	has a natural sense of rhythm	likes dancing	does the “runway” walk
	does drugs	has AIDS	is anorexic
	likes Fried Chicken	enjoys anal sex	enjoys musical theater
	works out a lot	speaks with his body language	is fashionable
	is aggressive	is emotionally expressive	is dramatic
	is threatening	is proud	is a good listener
	plays basketball for fun	spends time working for equal rights	likes going shopping
	is a criminal	doesn’t want children	is a nurse
	is a rapper	grew up without strong male role models	is a hairdresser
	is unintelligent	is secretive	has a lot of female friends
	is tall	is promiscuous	is an interior designer
	is uneducated	is friendly	is flamboyant
	is violent		is feminine
	is poor		wants his home to be stylish
	is good at sports		doesn’t like sports
	is athletic		is not athletic

#### Procedure

Each phrase was displayed on a half-sheet of paper as the only stimulus item in the thought-listing task (see samples in S1 File). Explicit tasks such as this one often bring in the concern that other psychological processes (e.g., censorship due to social desirability) may disrupt or distort the measurement of concept activation. Prior work has demonstrated, however, that these censorship concerns are mitigated by explicitly asking participants not to censor themselves, telling them we want their uncensored responses, and assuring them of their anonymity (e.g., 3, 27, 46–49). We employed these methods in the present work, and verified their effectiveness empirically in Study 2.

Written instructions directed participants to provide their gut responses and not to censor themselves, and they assured participants that all responses were completely anonymous. The instructions then prompted, “List the first five things that come to your mind when you picture:” followed by the stimulus phrase, in bold on the next line (e.g., “A man who is gay.”). Underneath the phrase were five numbered lines for the participants’ responses. At the bottom of the sheet were two items asking for participants’ gender and whether they had lived in the U.S. for more or fewer than 4 years.

Our task appeared at the end of a longer survey administered in introductory psychology classes. None of the other survey items or measures were relevant to prejudice, stereotyping, Black men, or gay men—this is important because if a survey item mentioned, for instance, Black men, that concept would be activated in participants’ minds, thus tainting our assessment of unidirectional associations. Each survey packet was identical in every way except for our thought-listing task, which contained only 1 stimulus item randomly selected from our list of items. Written consent was provided at the beginning of this survey, and an experimenter orally emphasized that we wanted uncensored responses and that the thought-listing task would be separated from the rest of the survey, thus reassuring anonymity.

#### Coding

Two pairs of independent coders who were blind to hypotheses coded every response in every condition. Each pair decided whether each response was one of the items in our study (e.g., *gay*) or a direct synonym of it (e.g., *homosexual*, *homo*, *faggot*). For example, if a participant’s responses were “homo,” “man-whore,” “HIV,” “stylish clothes,” and “effeminate,” we coded the participant’s responses as *gay*, *promiscuous*, *AIDS*, *fashionable*, and *feminine*, respectively. There was high interrater agreement (91.6%). Discrepancies between the pairs were resolved by discussion with an independent coder who was blind to condition, based purely on whether the response was a direct synonym of the concept in question. See S2 File for the raw participant responses and the results of the coding for Study 1.

### Results

#### Data and analysis description

**Fig 2:**
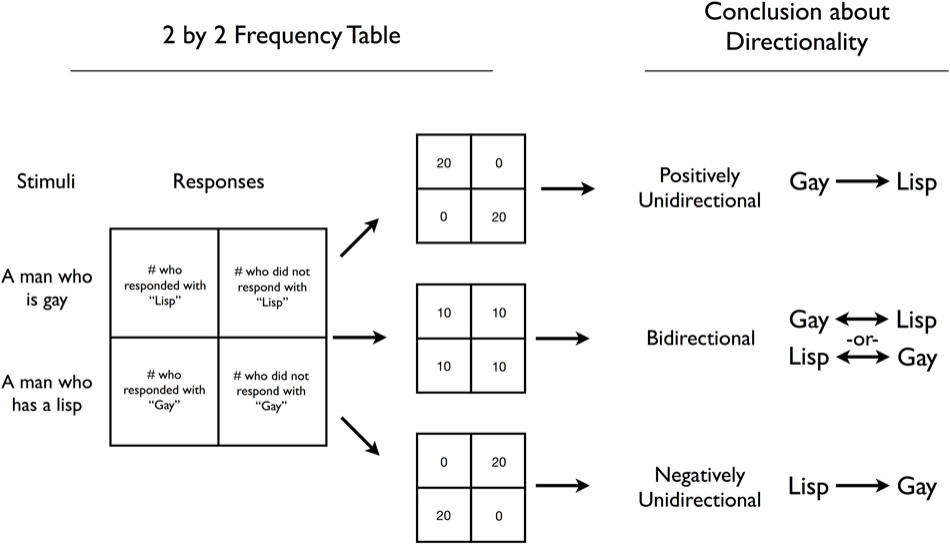
Experimental Design Flowchart. The directional structure of each stereotype link is assessed via a two-condition, between-subjects experiment. To test the *gay*–*lisp* stereotype, for example, participants are randomly assigned to produce associates for either *gay* or *lisp*. We count how many participants in each condition had this link activated (i.e., how many participants in the *gay* condition responded with *lisp* and how many participants in the *lisp* condition responded with *gay*). These counts are 1) submitted to a Yates’ chi-square test of independence, and 2) used to compute an effect size *d*. Yates’ chi-squares were calculated using [55], and logit-method d-scores and their 95% CIs were calculated using [56]. In the d-score calculations, we added 0.5 to each cell’s frequency count to avoid dividing by zero. Higher d-scores indicate more positive, Group → Attribute directionality, and lower d-scores indicate more negative, Attribute → Group directionality.

The coding yields a data matrix of association norms (S3 File), documenting how often each concept brought to mind each of the other concepts. For example, in the Black male condition, 20.5% of participants responded with *criminal*, 23.1% of participants responded with *athletic*, and 17.9% of participants responded with *poor*. We used these *inference percentages* to test the directionality of each Group—Attribute link, in what is essentially a two-condition experiment. For each link, we 1) conduct a Yates’ chi-square test of independence and 2) compute an effect size *d* that serves as a *directionality d-score*. Higher d-scores corresponded to more positive, Group → Attribute directionality, lower d-scores corresponded to negative, Attribute → Group directionality, and d-scores closer to zero corresponded to more bidirectionality. See Fig. 2 for a guide.

#### Stereotype directionality

As shown in Table 3, *Black* connected with 20 attributes. These links displayed heterogeneous directionality, with some having more positive directionality (e.g., *Black* → *poor*, *d* = 1.51), some being more evenly bidirectional (e.g., *Black* ⇄ *basketball*, *d* = 0.28), and some having more negative directionality (e.g., *AIDS* → *Black*, *d* = -1.99). *Gay* connected with 31 attributes (Table 4). These links also displayed heterogeneous directionality (e.g., g*ay* → *friendly*, *d* = 1.39; g*ay* ⇄ *speaks with body language*, *d* = -0.02; *hairdresser* → *gay*, *d* = -2.90).

**Table 3:** Directionality of Black Stereotype Links.

						Directionality		
		Percent of Participants For Whom		Chi-Square		d-score		
		the Stereotype Came to Mind		Statistics		95% CI		
	Attribute	Black→Attribute	Attribute→Black	Χ^2^_Yates_	*p*	Lower	Upper	*d*
**Black→Attribute**	Poor	17.9	0.0	4.675	0.031	-0.09	3.11	1.51
	Athletic	23.1	2.9	4.839	0.028	0.10	2.07	1.09
	Criminal	20.5	5.9	2.168	0.141	-0.13	1.52	0.69
	Tall	15.4	5.1	1.254	0.263	-0.26	1.43	0.59
	Basketball	17.9	11.1	0.260	0.610	-0.42	0.98	0.28
	Strong	5.1	2.8	0.269	0.604	-0.89	1.40	0.25
**Black←→Attribute**	Threatening	7.7	5.7	0.115	0.735	-0.79	1.07	0.14
	Good at sports	5.1	9.1	0.038	0.846	-1.23	0.63	-0.30
	Secretive	2.6	8.3	0.356	0.551	-1.63	0.54	-0.54
	Poor articulation	2.6	8.6	0.392	0.531	-1.65	0.53	-0.56
	Likes Hip Hop	12.8	35.3	3.954	0.050	-1.31	-0.06	-0.69
	Wears baggy clothing	5.1	32.4	7.690	0.006	-1.90	-0.30	-1.10
	Rapper	10.3	51.4	13.061	0.000	-1.82	-0.52	-1.17
	Does drugs	0.0	9.4	1.852	0.174	-2.89	0.42	-1.23
	Wears tank tops	0.0	9.4	1.852	0.174	-2.89	0.42	-1.23
	Natural rhythm	0.0	12.1	2.962	0.085	-3.00	0.26	-1.37
	Likes Fried Chicken	2.6	34.2	10.917	0.001	-2.41	-0.47	-1.44
	Beyoncé fan	0.0	20.6	6.665	0.010	-3.29	-0.09	-1.69
	Has AIDS	0.0	24.4	8.755	0.003	-3.39	-0.22	-1.80
**Attribute→Black**	Works for equal rights	0.0	31.3	11.721	0.001	-3.58	-0.40	-1.99

For clarity of presentation, in all the present studies, we excluded statistics for links that were activated for fewer than three participants. Including these weaker links does not alter the pattern of results, and they are reported fully in S2 and S3 Files. Numbers in the “Black **→** Attribute” column reflect the percentage of participants in the *Black* condition who responded with the given attribute. The “Attribute → Black” column contains the percentage of participants in that row’s attribute condition who responded with *Black*. In the test of the *Black*–*athletic* link, for example, 23.1% of participants in the *Black* condition responded with *athletic*, and 2.9% of the participants in the *athletic* condition responded with *Black*. The frequencies from which these percentages were calculated were submitted to Yates’ chi-square tests of independence that compare the likelihood that *Black* elicited the given attribute to the likelihood that the attribute elicited *Black*. The directionality d-scores derived from these frequencies provide an indicator of that stereotype link’s directionality. Higher, positive d-scores indicate that the link trends towards more positive, Group **→** Attribute directionality, and lower, negative d-scores indicate that the link trends towards more negative, Attribute **→** Group directionality.

**Table 4:** Directionality of Gay Stereotype Links.

						Directionality		
		Percent of Participants For Whom		Chi-Square		d-score		
		the Stereotype Came to Mind		Statistics		95% CI		
	Attribute	Gay→Attribute	Attribute→Gay	Χ^2^_Yates_	*p*	Lower	Upper	*d*
**Gay→Attribute**	Friendly	12.1	0.0	3.056	0.080	-0.25	3.02	1.39
	Well groomed	9.1	2.6	0.437	0.508	-0.51	1.67	0.58
	Well dressed	18.2	10.8	0.289	0.591	-0.41	1.03	0.31
**Gay←→Attribute**	Speaks w/ body language	6.1	6.3	0.001	0.975	-1.02	0.99	-0.02
	Fashionable	24.2	45.9	2.696	0.101	-1.07	0.04	-0.52
	Works for equal rights	3.0	12.5	0.935	0.334	-1.73	0.38	-0.68
	Uses hand gestures	3.0	13.9	1.372	0.241	-1.76	0.30	-0.73
	Has a lisp	3.0	17.1	2.294	0.130	-1.88	0.15	-0.86
	Flamboyant	12.1	48.7	9.393	0.002	-1.65	-0.36	-1.01
	Likes dancing	3.0	23.5	4.417	0.036	-2.07	-0.07	-1.07
	Feminine	15.2	57.9	11.939	0.001	-1.69	-0.46	-1.08
	Promiscuous	0.0	9.4	1.463	0.226	-2.80	0.51	-1.14
	Wears tight clothing	30.3	81.1	16.350	0.000	-1.81	-0.62	-1.22
	Doesn’t want children	0.0	11.1	2.124	0.145	-2.86	0.40	-1.23
	Doesn’t like sports	0.0	17.1	4.257	0.039	-3.09	0.12	-1.48
	Nurse	0.0	17.6	4.415	0.036	-3.11	0.11	-1.50
	Emotionally expressive	3.0	41.7	12.342	0.000	-2.49	-0.54	-1.52
	Anorexic	0.0	21.6	6.061	0.014	-3.23	-0.04	-1.63
	Dramatic	0.0	24.3	7.168	0.007	-3.30	-0.12	-1.71
	Has many female friends	6.1	65.6	22.672	0.000	-2.55	-0.93	-1.74
	Does the “runway” walk	3.0	56.7	19.602	0.000	-2.82	-0.86	-1.84
	Enjoys anal sex	6.1	69.7	25.756	0.000	-2.66	-1.03	-1.84
	Beyoncé fan	0.0	29.4	9.210	0.002	-3.44	-0.26	-1.85
	Likes shopping	3.0	62.5	23.623	0.000	-2.95	-0.99	-1.97
	Wears tank tops	0.0	43.8	15.903	0.000	-3.77	-0.60	-2.18
	Has AIDS	0.0	46.3	18.219	0.000	-3.81	-0.66	-2.24
	Interior designer	0.0	48.6	18.859	0.000	-3.87	-0.71	-2.29
	Enjoys musical theater	0.0	52.9	21.270	0.000	-3.96	-0.80	-2.38
	Wants a stylish home	0.0	58.1	23.864	0.000	-4.08	-0.90	-2.49
	Cher fan	0.0	60.0	24.858	0.000	-4.12	-0.94	-2.53
**Attribute→Gay**	Hair dresser	0.0	75.0	37.573	0.000	-4.50	-1.31	-2.90

In the “Gay **→** Attribute” column are the percentages of participants in the *Gay* condition who responded with the given attribute. Percentages in the “Attribute **→** Gay” column reflect the percent of participants in that row’s attribute condition who responded with *gay*. For example, 3.0% of participants given *gay* responded with *likes shopping*, and 62.5% of participants given *likes shopping* responded with *gay*. The chi-squares compare these likelihoods, and the directionality d-scores quantify the directionality of the links. Higher d-scores indicate more positive, Group **→** Attribute directionality, and lower d-scores indicate more negative, Attribute **→** Group directionality.

Meta-analytic techniques allow us to quantitatively combine the directionality scores of the individual links to provide a combined estimate of the stereotypes related to each group [57, 58]. At first glance, combining these effect sizes may seem statistically problematic because our design combines multiple outcomes (e.g., whether *gay* activated *fashionable* and also whether *gay* activated *promiscuous*) and uses multiple comparisons (e.g., participants in the *gay* condition are compared to participants in the *fashionable* condition and also to participants in the *promiscuous* condition). Nevertheless, such combinations are statistically sound for the computation of a single pooled effect size (although computing a CI for that effect size is more complex). See [58] for discussion of and equations for combining multiple outcomes (p. 226) and multiple comparisons (p. 240) into a single effect size.

**Fig 3:**
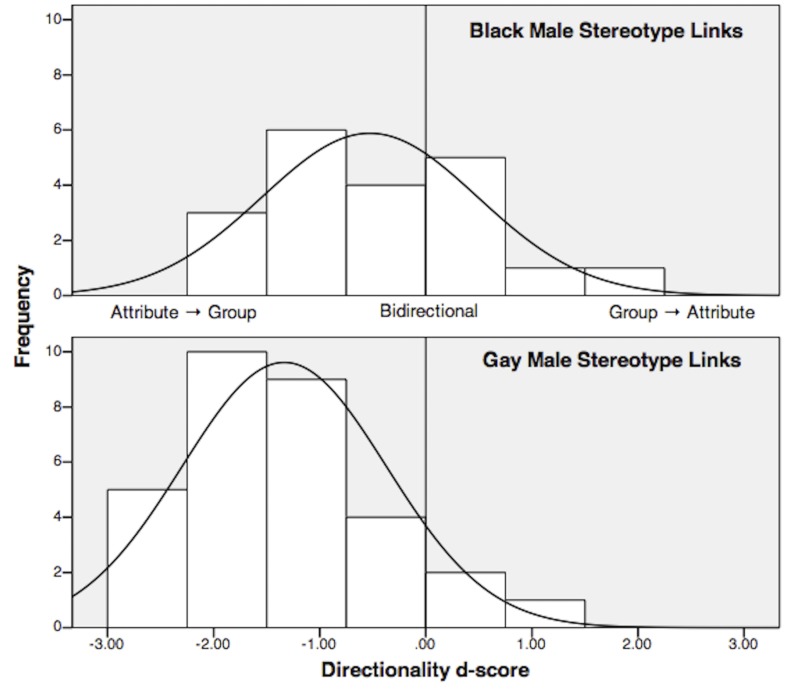
Study 1 Distributions of Directionality D-Scores. The gay stereotype links (*d*_*pooled*_ = -1.33; Bottom Panel) trended more strongly toward negative (Attribute → Group) directionality than the Black stereotype links (*d*_*pooled*_ = -0.53; Top Panel). Each set of directionality d-scores is normally distributed.

On the whole, the gay stereotypes trended toward negative directionality (*d*_*pooled*_ = -1.33) much more strongly than the Black stereotypes (*d*_*pooled*_ = -0.53), consistent with the argument that gay stereotypes often serve as categorization cues [28]. Further, as shown in Fig. 3, each group’s set of directionality d-scores was normally distributed, *Shapiro-Wilk*_*Black*_ (20) = 0.949, *p* = 0.359, skewness = 0.442, kurtosis = -0.852; *Shapiro-Wilk*_*Gay*_ (31) = 0.945, *p* = 0.111, skewness = 0.950, kurtosis = 1.054.

#### Directional network map

Due to space limitations, we only discuss connections with the primary nodes of interest (*gay* and *Black*) in-text. The provided data matrices, however, allow for a much more complex exploration of these stereotype networks and their underlying interconnected architecture. We also used these data to create an interactive visual map, which can be accessed at www.sciencecox.com/Map. This map provides a blueprint of the internal architecture of these stereotype networks.

### Discussion

Whereas prior stereotyping research has almost exclusively emphasized positive, Group → Attribute unidirectionality, Study 1 revealed stereotypes with highly heterogeneous directionality (d-score range: -2.90 to 1.51), including negative, Attribute → Group unidirectionality and bidirectionality (Attribute ⇄ Group). This heterogeneity matches our predictions, and would not have arisen as easily from traditional models, which most often define stereotyping as a process that occurs after group membership activation.

Participants were highly likely to make both *Black* → *basketball* and *basketball* → *Black* inferences, corroborating prior work’s conclusions about this stereotype being bidirectional [29]. Also, the gay stereotype links overall tended towards negative directionality (Attribute → Group), corroborating work showing that gay male stereotypes are used to make inferences about group membership [27–28, 59]. We argue that this negative directionality likely reflects how these stereotypes developed and are used in society to differentiate and identify gay men. Indeed, many of the modern stereotypes of gay men can be traced to media portrayals in the 1930’s, when U.S. movies were banned from showing homosexual characters. As a way around these bans, many movies included male characters whose orientation was never stated, but was implied by effeminate behavior, flamboyant dress, lisped speech, or other stereotypic traits [59]. The cultural development and social functions of these stereotypes, we argue, are reflected in their patterns of directionality. Because people often rely on gay stereotypes to infer group membership (e.g., seeing that a man wears tank tops is used to infer that he is gay), the gay stereotypes more often have negative, Attribute → Group directionality [28].

Because our measure relied on self-report, it is possible that participants censored socially undesirable responses. Further, it is possible that the social desirability of a response differs by the direction of an inference (e.g., perhaps it is more socially unacceptable to infer that a criminal man is Black than that a Black man is criminal). To the extent that the inference percentages of Study 1 are influenced by social desirability concerns (which may themselves have heterogeneous directionality), our conclusions about stereotype directionality would be limited. We took care to implement methodological procedures shown to mitigate socially desirability pressures on this sort of measure. Further, other work has shown that stereotypic responses on this measure are unrelated to the internal and external motivations to respond without prejudice, two well-validated measures that strongly predict participants’ likelihood to self-censor or hide stereotypic responses [27]. For an added level of scrutiny, however, in Study 2 we directly assessed the extent to which the social acceptability of a given inference relates to the probability that participants reported that inference.

## Study 2

### Method

As a part of a different study examining the directionality of normative pressures, 42 undergraduate participants were asked to rate how acceptable various inferences were, according to societal standards. Each participant rated 63 inferences total, and 26 of those corresponded to the inferences in Study 1. The instructions read:

“In life, people make many different inferences/assumptions about others. Sometimes these inferences are acceptable and appropriate, and sometimes the inferences are unacceptable and inappropriate. To determine if an inference is acceptable or unacceptable, it’s important to have a standard in mind (i.e., acceptable or unacceptable according to who?).

In what follows, we will present you with a series of inferences to consider. As you consider them, we would like you think about society’s values and how acceptable SOCIETY believes each of the following inferences is. On the following screens, you will rate each inference by moving a slider toward "strongly disagree" or "strongly agree" to indicate how much you agree the inference is acceptable according to SOCIETAL values. Some of the items will be similar, but you should consider each item independently.”

At the top of each page of the online survey, the task prompted, “According to SOCIETAL VALUES…” with three inferences underneath for participants to rate.

Participants were randomly assigned to one of two “lists” of inferences. Each list contained a mix of inferences related to Black men and gay men, and a mix of inferences in the Group → Attribute direction and the Attribute → Group direction. Each group—attribute pair appeared only once in each list, and the inferences of the two lists were in the opposite direction of each other. If, for instance, List 1 contained the item, “If I find out that a man is gay, it is acceptable to think that he probably is fashionable” (thus rating the acceptability of *gay* → *fashionable*), then List 2 would have the opposite, “If I find out that a man is fashionable, it is acceptable to think that he probably is gay” (thus rating the acceptability of *fashionable* → *gay*). Participants rated their agreement with these statements using a slider (0 = *Strongly Disagree* to 100 = *Strongly Agree*). We used the means of these ratings as *social acceptability scores*, which we compare to the corresponding inference percentages of Study 1.

### Results and Discussion

The social acceptability scores and their corresponding inference percentages from Study 1 are displayed in Table 5. Overall, there appears to be no systematic difference in social acceptability based on whether the inference occurs in the Group → Attribute or Attribute → Group direction, *t* (50) = 0.890, *p* = 0.378. Also, the acceptability of the Group → Attribute inferences is unrelated to the acceptability of their corresponding Attribute → Group inferences, *r* = 0.060, *p* = 0.771.

**Table 5:** Social Acceptability Ratings in Study 2.

Gay Male Stereotypes			Black Male Stereotypes		
Inference	Study 1 Inference Percentage	Study 2 Acceptability Mean (SD)	Inference	Study 1 Inference Percentage	Inference
AIDS→Gay	46.3	23.4 (23.44)	Articulation→Black	8.6	46.6 (19.61)
Gay→AIDS	0.0	31.7 (17.70)	Black→Articulation	2.6	30.3 (25.15)
FemFriends→Gay	65.6	54.1 (24.92)	BaggyClothes→Black	32.4	40.9 (24.67)
Gay→FemFriends	6.1	41.0 (18.87)	Black→BaggyClothes	5.1	34.8 (25.93)
Feminine→Gay	57.9	52.6 (32.09)	Chicken→Black	34.2	49.7 (31.01)
Gay→Feminine	15.2	59.9 (19.89)	Black→Chicken	2.6	31.0 (28.11)
Dancing→Gay	23.5	54.7 (25.45)	Poor→Black	0.0	45.2 (18.94)
Gay→Dancing	3.0	30.1 (23.58)	Black→Poor	18.0	32.0 (25.36)
Designer→Gay	48.6	38.5 (14.42)	Strong→Black	2.8	54.0 (20.51)
Gay→Designer	0.0	48.1 (30.01)	Black→Strong	5.1	24.7 (20.70)
Fashionable→Gay	46.0	60.8 (26.27)	Tall→Black	5.1	49.8 (17.89)
Gay→Fashionable	24.2	42.7 (24.92)	Black→Tall	15.4	25.5 (21.16)
Flamboyant→Gay	48.7	52.5 (22.36)	Criminal→Black	5.9	30.1 (26.96)
Gay→Flamboyant	12.1	52.4 (29.83)	Black→Criminal	20.5	46.8 (23.14)
Hairdresser→Gay	75.0	32.3 (19.35)	Rapper→Black	51.4	33.6 (25.64)
Gay→Hairdresser	0.0	49.7 (30.72)	Black→Rapper	10.3	69.2 (17.07)
NotLikeSports→Gay	17.1	43.8 (19.02)	Sports→Black	9.1	53.0 (30.36)
Gay→NotLikeSports	0.0	34.7 (28.23)	Black→Sports	5.1	56.9 (14.43)
Promiscuous→Gay	9.4	36.0 (26.41)	Threatening→Black	5.7	37.6 (27.87)
Gay→Promiscuous	0.0	26.9 (23.05)	Black→Threatening	7.7	46.5 (15.66)
StylishHome→Gay	58.1	38.6 (24.05)			
Gay→StylishHome	0.0	47.0 (25.33)			
NotWantChildren→Gay	11.1	24.7 (23.55)			
Gay→NotWantChildren	0.0	25.4 (15.95)			
Shopping→Gay	62.5	44.2 (28.90)			
Gay→Shopping	3.0	50.1 (17.53)			
WellDressed→Gay	10.8	51.6 (29.34)			
Gay→WellDressed	18.2	43.4 (20.18)			

Numbers in the “Study 1 Inference Percentage” column reflect the percent of participants who made that inference in Study 1. Study 2’s social acceptability scores were obtained by participants rating their agreement with statements such as “According to societal standards … If I find out that a man is gay, it is acceptable to think that he probably is fashionable” using a slider (0 = *Strongly Disagree* to 100 = *Strongly Agree*).

The inferences’ social acceptability scores were completely unrelated to their Study 1 inference percentages, *r* = 0.083, *p* = 0.561. Thus, it seems that the methods of Study 1 were effective at mitigating socially desirable responding: normative concerns do not provide a compelling account for Study 1’s findings. In Study 3, we address another alternate explanation that suggests the heterogeneity found in Study 1 may be an artifact of random chance.

## Study 3

A skeptic might argue that the normal distributions of each set of directionality d-scores (see again Fig. 3) indicate that the stereotypes are not separate links with heterogeneous directionality. It is also possible that these normal distributions reflect chance variations around a single shared mean, and that the extreme scores would regress towards that mean in a new sample, washing out the heterogeneity. This interpretation is consistent with, for example, prototype models that conceptualize a stereotype as a single, composite representation. We argue, nevertheless, that the normal distributions reflect actual normal distributions of the separate, component stereotype links. The key to resolving these opposing perspectives is replication with a larger sample size. If the heterogeneity found in Study 1 occurred due to chance variations around a single mean, then a second measurement of the same links with a larger sample should yield directionality scores that regress toward that single, composite mean. If each link indeed has its own directionality, however, that link’s directionality d-score should be roughly the same in a second test and fall within the 95% confidence intervals (CIs) of the original score. Further, a replication with a larger sample size will provide a more accurate, stable estimate of each link’s directionality, with tighter 95% CIs. In Study 3, therefore, we selected six prominent stereotype links from Study 1 and replicated their assessment with more people assigned to each condition.

### Method

The procedure was identical to Study 1, except participants were each randomly assigned to one of only eight conditions (*Black*, *athletic*, *criminal*, *poor*, *gay*, *flamboyant*, *fashionable*, *dramatic*), which correspond to six stereotype links of interest (*Black*–*athletic*, *Black*–*criminal*, *Black*–*poor*, *gay*–*flamboyant*, *gay*–*fashionable*, *gay*–*dramatic*). After exclusions (*Non-U*.*S*. = 76; *Missing more than 1 data point* = 37), Study 3 had 1342 participants total, enabling us to assess the directionality of each stereotype link with greater power (1-β = 0.99 for *d* = 0.3). Raw participant data and coding are provided in S4 File, and the resulting data matrix is in S3 File.

### Results and Discussion

The Study 3 analyses of each link and comparisons to their Study 1 counterparts are displayed in Table 6. Each Study 3 directionality d-score fits perfectly within the 95% CI of its Study 1 counterpart, indicating that Study 1’s conclusions about heterogeneity did not merely capitalize on random chance or smaller per-condition sample sizes. Study 3’s patterns give us greater confidence in the stability of the specific directionality d-scores obtained for each link in Study 1, and in our conclusions about heterogeneous directionality overall. Further, note that the 95% CIs for the *gay*–*fashionable* link and the *gay*–*flamboyant* link have no overlap, and the CIs of the *Black*–*athletic* link has no overlap with the CIs of the *Black*—*criminal* link. Because there is no overlap in these CIs, we can conclude that these stereotype links have truly distinct directionality. This replication validates our conclusions about the heterogeneous directionality of stereotype links: each link has its own particular directional structure. Study 3 also validates our experimental paradigm and methodology more generally, showing that given links’ directionality d-scores replicate with new, much larger samples in each condition.

**Table 6:** Directionality of Study 3 Stereotype Links and Comparison to Same Links in Study 1.

		Percent of Participants For Whom		Chi-Square		Study 3 d-score			Study 1 d-score		
		the Stereotype Came to Mind		Statistics		95% CI			95% CI		
Group	Attribute	Group→Attribute	Attribute→Group	χ^2^_Yates_	*p*	Lower	Upper	*d*	Lower	Upper	*d*
**Black**	Athletic	36.0	4.4	47.486	0.000	0.91	1.79	1.35	0.10	2.07	1.09
	Poor	11.8	5.0	3.976	0.046	0.03	0.96	0.50	-0.09	3.11	1.51
	Criminal	15.5	10.7	1.327	0.249	-0.12	0.59	0.23	-0.13	1.52	0.69
**Gay**	Fashionable	25.0	41.5	8.929	0.003	-0.68	-0.15	-0.41	-1.07	0.04	-0.52
	Dramatic	5.8	25.6	21.789	0.000	-1.34	-0.52	-0.93	-3.30	-0.12	-1.71
	Flamboyant	14.7	51.7	48.173	0.000	-1.29	-0.70	-1.00	-1.65	-0.36	-1.01

In the “Group **→** Attribute” column are the percentages of participants in the given group condition who responded with the given attribute. Percentages in the “Attribute **→** Group” column reflect the percent of participants in that row’s attribute condition who responded with to corresponding group. For example, 36.0% of participants given *Black* responded with *athletic*, and 4.4% of participants given *athletic* responded with *Black*. The chi-squares compare these likelihoods, and the directionality d-scores quantify the directionality of the links. Higher d-scores indicate more positive, Group **→** Attribute directionality, and lower d-scores indicate more negative, Attribute **→** Group directionality.

Studies 1 and 3 provide ample support for heterogeneous stereotype directionality. Stereotypes are not limited to the positive, Group → Attribute direction emphasized by traditional models. Further, individual stereotype links have their own particular directionality, varying both among the links related to a single group and across the networks of different stereotyped groups. A limitation of Studies 1 and 3, however, is that they only examined stereotypes related to Black men and gay men. Although these are two of the most-studied social groups within the prejudice and stereotyping literature, they may differ from other stereotyped groups of interest. In Study 4, therefore, we extend our exploration to a wide variety of other social groups.

## Study 4

Whereas Study 1 explored the stereotype networks of two social groups in depth, in Study 4 we exchanged depth in two groups for breadth across a sampling of several groups that have been of interest to social psychologists historically (e.g., *Jewish people*) and contemporarily (e.g., *Arab-Muslim people*). Study 4 allowed us to demonstrate that heterogeneous stereotype directionality is not limited to the particular stereotypes we selected in Study 1 and provides another set of data matrices in which to explore stereotype structure.

### Method

The procedure was identical to Study 1, with undergraduates being randomly assigned to one of 60 conditions. After exclusions (*Non-U*.*S*. = 150; *Missing more than 1 data point* = 60), we had 1384 participants total. We assessed a wide variety of stereotyped social concepts (e.g., *lesbians*, *Jewish people*, *White people*). See the full list of stimuli in Table 7. To maximize our efficiency, we made efforts to select concepts stereotypically related to multiple potential groups of interest (e.g., *stingy* is stereotypically linked to Republicans and Jews; *anti-gay* to Arab-Muslims, Catholics, and Republicans). The raw data are provided in S5 File (“Person” conditions) and S6 File (“Woman” conditions). We chose seven prominent social groups to explore in-text as representatives of Study 4’s data (*Lesbians*, *Jews*, *Arab-Muslims*, *Catholics*, *Whites*, *Democrats*, and *Republicans*), but the full data matrixes, in S3 File, contain many other potential groups of interest (e.g., *Europeans*, *Southerners*). These selected groups provide a broad sampling with which to demonstrate heterogeneous directionality in-text.

**Table 7:** Stimulus Phrases for Study 4.

**A woman who…**			
	doesn't wear makeup	is a lesbian	plays softball
	has short hair	is a tomboy	wears cut off tees
	is a construction worker	is a women's rights activist	
**A person who…**			
	is Jewish	is an Evangelical Christian	is a Coastie^a^
	is Arab-Muslim	is in a Fraternity	wears Ugg boots
	is white	is in a Sorority	is open-minded
	is Catholic	is a CEO	is poor
	is a Conservative/Republican	is European	is religious
	is a Liberal/Democrat	is from the South	is stingy
	drinks alcohol	is Atheist	is strict
	has a beard	is blonde	is stuck up
	has a big nose	is close-minded	is traditional
	has an accent	is confrontational	parties a lot
	has expensive things	is ditzy	smokes marijuana
	has many children	is educated	smokes
	is judgmental	is evil	is mean
	is an activist	is free-thinking	is not hard-working
	is an environmentalist	is hard-working	is anti-gay
	thinks they are superior to others		is arrogant
	has parents that make a lot of money		
	is very devoted to their beliefs		
	marries within their own culture/religion		
	is intolerant of other cultures/religions		
	is ignorant of Wisconsin culture		

These are the stimulus items for Study 4.

^a^ *Coastie* is a slang term that many native Wisconsin students (i.e., *Sconnies*) at the University of Wisconsin use to derogate students from out-of-state, specifically those from the East and West coasts of the United States. We selected this group because there had been several hot-button campus incidents involving anti-Coastie prejudice around the time these data were collected [60].

### Results and Discussion

There was heterogeneous directionality within and between all of the stereotype networks. See Table 8 for stereotypes related to lesbian women, Jewish people, and Arab-Muslim people. The lesbian stereotypes, like gay male stereotypes, trended overall towards negative directionality (*d*_*pooled*_ = -0.84). Although, like gay men and lesbian women, Jewish people lack visible defining features, the Jewish stereotypes trended more towards positive directionality (*d*_*pooled*_ = 0.71), perhaps indicating that Jewish stereotypes did not develop primarily to aid inferences about group membership (for further discussion of the cultural development of stereotypes, see [61–62]). Nevertheless, some of the Jewish stereotypes did have high probabilities of being activated in the Attribute → Group direction, indicating that they could be used to infer group membership (e.g., *Big Nose* → *Jew*; of the people who received “A person with a big nose” as a stimulus, 50% responded with “Jew”). The Arab-Muslim stereotypes were also heterogeneous, but trended towards positive directionality overall (*d*_*pooled*_ = 0.90). As shown in Table 9, both the Catholic (*d*_*pooled*_ = -0.08) and White (*d*_*pooled*_ = -0.03) stereotypes had diverse, normally distributed directionality scores centered around *d* = 0. Lastly, the stereotypes about liberals/democrats (*d*_*pooled*_ = -0.23) and conservatives/republicans (*d*_*pooled*_ = 0.61) are displayed in Table 10.

**Table 8:** Directionality of Lesbian, Jew, and Arab Stereotype Links.

						Directionality		
		Percent of Participants For Whom		Chi-Square		d-score		
		the Stereotype Came to Mind		Statistics		95% CI		
Group	Attribute	Group→Attribute	Attribute→Group	Χ^2^_Yates_	*p*	Lower	Upper	*d*
**Lesbians**	Tomboy	69.2	45.8	1.926	0.165	-0.11	1.14	0.52
	Women’s rights activist	3.8	7.1	0.279	0.597	-1.42	0.90	-0.26
	Has short hair	46.2	61.9	0.611	0.434	-0.97	0.29	-0.34
	Plays softball	7.7	57.7	12.585	0.000	-2.25	-0.59	-1.42
	Wears cutoff tees	0.0	29.6	6.909	0.009	-3.34	-0.13	-1.73
	Construction Worker	0.0	31.6	6.938	0.008	-3.41	-0.16	-1.79
						dPooled = -0.84		
**Jews**	Has wealthy parents	26.1	0.0	4.322	0.038	-0.10	3.15	1.53
	White	17.4	0.0	2.602	0.107	-0.31	2.98	1.34
	Religious	56.5	20.0	5.348	0.021	0.18	1.55	0.86
	Has a beard	8.7	6.7	0.077	0.782	-0.86	1.17	0.16
	Big nose	34.8	50.0	0.438	0.508	-1.01	0.35	-0.33
						dPooled = 0.71		
**Arabs**	Confrontational	44.8	0.0	10.498	0.001	0.37	3.56	1.96
	Mean	41.4	0.0	9.278	0.002	0.29	3.49	1.89
	Religious	27.6	0.0	6.058	0.014	0.05	3.26	1.66
	Evil	20.7	0.0	3.172	0.075	-0.25	2.98	1.37
	Has a beard	17.2	3.3	1.785	0.182	-0.22	1.86	0.82
	Accent	10.3	15.0	0.002	0.964	-1.11	0.65	-0.23
	Activist	0.0	10.3	1.406	0.236	-2.79	0.53	-1.13
						dPooled = 0.90		

In the “Group **→** Attribute” column are the percentages of participants in the given group condition who responded with the given attribute. Percentages in the “Attribute **→** Group” column reflect the percent of participants in that row’s attribute condition who responded with to corresponding group. The chi-squares compare these likelihoods, and the directionality d-scores quantify the directionality of the links. Higher d-scores indicate more positive, Group **→** Attribute directionality, and lower d-scores indicate more negative, Attribute **→** Group directionality.

**Table 9:** Directionality of Catholic and White Stereotype Links.

						Directionality		
		Percent of Participants For Whom		Chi-Square		d-score		
		the Stereotype Came to Mind		Statistics		95% CI		
Group	Attribute	Group→Attribute	Attribute→Group	Χ^2^_Yates_	*p*	Lower	Upper	*d*
**Catholics**	Strict	27.3	0.0	4.331	0.037	-0.09	3.16	1.53
	White	18.2	0.0	2.764	0.096	-0.28	3.01	1.37
	Close-minded	13.6	0.0	1.622	0.203	-0.47	2.87	1.20
	Has many children	18.2	8.0	0.367	0.545	-0.46	1.37	0.46
	Traditional	9.1	4.5	0.358	0.550	-0.86	1.47	0.31
	Religious	31.8	36.0	0.091	0.763	-0.75	0.56	-0.10
	Intolerant of other cultures	9.1	19.0	0.252	0.616	-1.33	0.51	-0.41
	Conservative/Republican	4.5	14.8	0.500	0.480	-1.63	0.51	-0.56
	Anti-Gay	0.0	14.3	1.751	0.186	-2.81	0.48	-1.16
	Devoted to beliefs	0.0	33.3	6.484	0.011	-3.36	-0.12	-1.74
	Marries within own culture	0.0	35.3	6.666	0.010	-3.42	-0.15	-1.78
						dPooled = -0.08		
**Whites**	Stuck up	29.2	0.0	5.203	0.023	-0.01	3.22	1.61
	Corporate	41.7	4.8	6.382	0.012	0.25	2.28	1.26
	Close-minded	12.5	0.0	1.422	0.233	-0.52	2.81	1.14
	Confrontational	12.5	0.0	1.162	0.281	-0.59	2.74	1.07
	Hard-working	25.0	4.2	2.676	0.102	-0.09	1.98	0.94
	Educated	37.5	9.5	3.352	0.067	0.01	1.71	0.86
	Arrogant	20.8	4.8	1.306	0.253	-0.31	1.80	0.74
	Has expensive things	25.0	8.0	1.495	0.221	-0.21	1.53	0.66
	Blonde	8.3	9.1	0.008	0.927	-1.07	0.97	-0.05
	Thinks they are superior	4.2	11.1	0.227	0.634	-1.49	0.63	-0.43
	Intolerant of other cultures	4.2	19.0	1.230	0.267	-1.84	0.31	-0.77
	Anti-Gay	0.0	10.7	1.114	0.291	-2.71	0.61	-1.05
	Conservative/Republican	0.0	11.1	1.182	0.277	-2.74	0.59	-1.07
	From the South of the U.S.	0.0	14.3	1.736	0.188	-2.90	0.44	-1.23
	Jewish	0.0	17.4	2.602	0.107	-2.98	-3.28	-1.34
	Catholic	0.0	18.2	2.764	0.096	-3.01	0.28	-1.37
	Marries within own culture	0.0	23.5	3.870	0.049	-3.19	0.11	-1.54
						dPooled = -0.03		

In the “Group **→** Attribute” column are the percentages of participants in the given group condition who responded with the given attribute. Percentages in the “Attribute **→** Group” column reflect the percent of participants in that row’s attribute condition who responded with to corresponding group. The chi-squares compare these likelihoods, and the directionality d-scores quantify the directionality of the links. Higher d-scores indicate more positive, Group **→** Attribute directionality, and lower d-scores indicate more negative, Attribute **→** Group directionality.

**Table 10:** Directionality of Democrat and Republican Stereotype Links.

						Directionality		
		Percent of Participants For Whom		Chi-Square		d-score		
		the Stereotype Came to Mind		Statistics		95% CI		
Group	Attribute	Group→Attribute	Attribute→Group	Χ^2^_Yates_	*p*	Lower	Upper	*d*
**Democrats**	Conservative/Republican	13.6	0.0	1.908	0.167	-0.40	2.93	1.26
	Open-minded	22.7	19.0	0.088	0.767	-0.67	0.89	0.11
	Environmentalist	13.6	20.0	0.052	0.819	-1.00	0.57	-0.22
	Thinks they are superior	4.5	8.3	0.000	0.985	-1.32	0.87	-0.22
	Activist	22.7	41.4	1.209	0.272	-1.12	0.21	-0.45
	Free-thinking	18.2	36.4	1.031	0.310	-1.22	0.25	-0.49
	Smokes marijuana	4.5	19.0	1.014	0.314	-1.80	0.36	-0.72
	Atheist	0.0	13.6	1.431	0.232	-2.82	0.52	-1.15
						dPooled = -0.23		
**Republicans**	Has expensive things	37.0	0.0	9.203	0.002	0.29	3.49	1.89
	Judgmental	25.9	0.0	5.430	0.020	0.00	3.22	1.61
	Thinks they are superior	18.5	0.0	4.929	0.026	-0.03	3.21	1.59
	Stuck up	25.9	0.0	4.463	0.035	-0.09	3.13	1.52
	Mean	22.2	0.0	3.495	0.062	-0.21	3.03	1.41
	Arrogant	18.5	0.0	2.583	0.108	-0.33	2.93	1.30
	Educated	14.8	0.0	1.732	0.188	-0.48	2.81	1.16
	Corporate	14.8	0.0	1.732	0.188	-0.48	2.81	1.16
	Close-minded	33.3	4.2	5.132	0.024	0.14	2.16	1.15
	White	11.1	0.0	1.182	0.277	-0.59	2.74	1.07
	Religious	40.7	16.0	2.760	0.097	-0.03	1.36	0.66
	Catholic	14.8	4.5	0.500	0.480	-0.51	1.63	0.56
	Intolerant of other cultures	33.3	19.0	0.605	0.437	-0.33	1.10	0.38
	Confrontational	11.1	4.8	0.069	0.792	-0.73	1.47	0.37
	Wealthy Parents	11.1	4.8	0.069	0.792	-0.73	1.47	0.37
	Evangelical Christian	7.4	3.4	0.004	0.949	-0.81	1.50	0.34
	Activist	18.5	10.3	0.241	0.623	-0.46	1.14	0.34
	Strict	7.4	10.0	0.099	0.753	-1.20	0.84	-0.18
	From the South	14.8	23.8	0.176	0.675	-1.08	0.46	-0.31
	Traditional	18.5	36.4	1.171	0.279	-1.18	0.21	-0.48
	Liberal/Democrat	0.0	13.6	1.908	0.167	-2.93	0.40	-1.26
	Anti-gay	7.4	46.4	8.676	0.003	-2.03	-0.38	-1.20
						dPooled = 0.61		

In the “Group **→** Attribute” column are the percentages of participants in the given group condition who responded with the given attribute. Percentages in the “Attribute **→** Group” column reflect the percent of participants in that row’s attribute condition who responded with to corresponding group. The chi-squares compare these likelihoods, and the directionality d-scores quantify the directionality of the links. Higher d-scores indicate more positive, Group **→** Attribute directionality, and lower d-scores indicate more negative, Attribute **→** Group directionality.

In sum, Study 4 found diverse, heterogeneous varieties of directionality, replicating Study 1 with various stereotypes and groups. Rather than being an artifact of Black male or gay male stereotypes, heterogeneous stereotype directionality occurs within and between the stereotype links related to many different groups.

## General Discussion

The present work provided ample evidence that stereotypes possess heterogeneous directionality. Our prediction of heterogeneous stereotype directionality would not arise readily from traditional models of stereotyping, which either directly or tacitly make assumptions that stereotyping operates in the positive, Group → Attribute direction. According to our model, directionality is a fundamental feature of stereotypes and stereotyping. Although largely unnoticed and unexplored in the literature, stereotype directionality invites many new questions and has a wide range of theoretical and empirical implications for stereotyping and prejudice research and theory. In what follows, we discuss possible contributors to directionality, implications of directionality for behavior, regulation, and change, and some ways that theoretical consideration of directionality may alter how we interpret various measures of associations. We then outline some of the possible uses of the data matrices from the present work and discuss our connectionist-informed approach to stereotypes and stereotyping.

### Contributors to Directionality

Just as there are a nearly infinite number of factors that can influence how strong an association is, how easily it is activated, and how it came to be learned, so too should there be an abundance of factors that determines an association’s directionality. Study 1 suggests that the relative visibility of a social concept is an important contributor to directionality, and there are likely many others to be identified. For any given stereotype, each connected concept has many features (e.g., visibility, entitativity, concept crowdedness) that should contribute to the association’s directionality. Each concept in memory, for instance, has its own level of *concept crowdedness*, which refers to the density or sparsity of the concept space, (i.e., how many or few associates that concept brings to mind, see [35]). If concept A has only a single associate, B, but concept B has many associates, there will be a greater likelihood that stimulus A will elicit response B than the reverse. The relative crowdedness of each linked concept must contribute to the likelihood that the other will come to mind, and the relationship between these likelihoods is, by definition, directionality.

Directionality should also reflect the way in which the stereotype is learned, reinforced, and used [34–35]. For example, as we suggested in Study 1, when the defining features of group membership are non-visible, as is the case with gay men, stereotypes may develop that link group membership to visible attributes that can serve as categorization cues [28]. These stereotypes make group membership ostensibly visible, and relying on stereotypic attributes to infer group membership should build associations primarily from the attribute to the group, leading to observed Attribute → Group directionality. Understanding a stereotype’s directionality, therefore, may give us insight into the social functions or origins of that stereotype. In this way, our exploration of directionality provides a foundation from which to generate hypotheses regarding the multifarious contributors to directionality, and in so doing, we can better understand stereotypes and stereotyping.

### Implications for Behavior, Regulation, and Change

For any examination of stereotypes and stereotyping, we strongly recommend considering the directionality of the specific stereotype under study. The heterogeneous directionality both within and between the stereotype networks of different social groups suggests that phenomena demonstrated with one stereotype link (e.g., *Black* → *athletic*) or one stereotyped group (e.g., *Black men*) may not generalize to other links (e.g., *Black* ⇄ *basketball*) or groups (e.g., *gay men*) that have different patterns of directionality. And, indeed, stereotypes with different directionality bring in different sets of theoretical and practical concerns.

The consequences of Group → Attribute stereotyping are well-documented: The activation of a stigmatized group concept brings to mind an assortment of stereotypic attributes that influence the perceiver’s judgments, attention, affect, and behavior towards the group member, usually to the target’s detriment (for a review, see [2]). Consider now the consequences of Attribute → Group stereotypes. First and foremost, once a stereotypic attribute activates the group status, that activation will spread through all the Group → Attribute connections as well, setting off the same negative consequences that result from the typical case of Group → Attribute stereotyping.

Attribute → Group stereotyping also has additional, unsettling implications for the expression of prejudice [27]. Suppose a prejudice perpetrator uses a stereotypic attribute to make an assumption about their victim’s group status, and subsequently expresses prejudice (e.g., aggression) based on that inferred group membership. Because the stereotypic assumption can remain private in the perpetrator’s mind, he or she can later deny that prejudice motivated his or her actions. Thus, Attribute → Group stereotypes can grant prejudice perpetrators “plausible deniability” for their prejudice, freeing them from concerns about appearing prejudiced to others [27]. Bidirectional stereotypes, lastly, bring in all of the above concerns, because they involve both Group → Attribute and Attribute → Group inferences. These bidirectional stereotypes may also be especially easy to reinforce and hard to change because the activation of either concept will bring to mind the other. This reciprocal activation potentially doubles the opportunities for the stereotype to be activated and reinforced.

Directionality may be especially important to take into account for stereotype change efforts. Most existing stereotype change methods focus on Group → Attribute stereotyping, which may be sufficient for stereotypes that are themselves primarily Group → Attribute unidirectional. For a bidirectional stereotype like *Black* ⇄ *criminal*, however, further considerations come into play. If an intervention changes *Black* → *criminal* associations or teaches people to inhibit or otherwise regulate *Black* → *criminal* inferences, it likely leaves untouched the *criminal* → *Black* associations and inferences. Even if people reject the notion that most Black people are criminals, they may still think most criminals are Black. These remaining *criminal* → *Black* associations can have important consequences in contexts where *criminal* is activated on its own, as in police lineups, jury deliberations [63] or split-second shooting decisions [64–65]. To be fully effective, an intervention against a bidirectional stereotype may need to target both nodes (see also [29, 66]). Likewise, purely Group → Attribute interventions may be ineffective against stereotypes that are primarily Attribute → Group. If people are motivated to refrain from assuming that gay men are feminine, that does not necessarily stop them from stereotyping feminine men as gay—and they may not even recognize the latter as stereotyping [28]. Full consideration of directionality will focus theorists and researchers more directly on the best avenues for understanding stereotype development, reinforcement, and change processes.

### Implications for Measure Interpretation and Validity

Consideration of directionality leads one to think differently about how we interpret various measures. Consider implicit reaction time measures, which are popular for the study of stereotype activation. A sequential priming task, for example, is most commonly interpreted as measuring the Group → Attribute association/activation strength, because it can be set up in a way that seems unidirectional at the level of a single trial (e.g., participants see the prime “Black” and respond to the target “athletic”). Consideration of directionality, however, may call this measure’s interpretation into question. Reaction time measures require many trials to get stable estimates of response latencies [67]. Because the first trial activates both of the concepts, both concepts are active in memory *before* the onset of subsequent trials. After the first trial, therefore, it is unclear whether shorter response latencies arise from associations in one direction versus the other. Thus, the task as a whole is inherently bidirectional, even if a single trial seems unidirectional. Unless we could rely on a single trial or assume that activation completely fades between trials, it seems to us that reaction time measures cannot cleanly assess unidirectional structure.

Because traditional models disregard directionality, current measures of association strength sometimes reflect the bidirectional association strength (i.e., Group → Attribute + Attribute → Group associations, as in most reaction time measures) and sometimes reflect the positively unidirectional association strength (i.e., Group → Attribute associations, as in thought-listing tasks). Depending on one’s experimental design and specific research question, using a bidirectional measure may pose no problem, or it may highly distort one’s assessment of the target constructs and processes. To illustrate, suppose that a researcher uses sequential priming tasks to assess the relative strength of three stereotypic associations, A–B, A–C, and A–D. Suppose A–B is strongly bidirectional, with A → B and B → A each having a weight of 5. Association A–C, however, is positively unidirectional, with A → C having a weight of 10, and no association from C to A. Lastly, A–D is negatively unidirectional, with no association from A to D, but with D → A having a weight of 10. For each of these associations, the (bidirectional) association strength, as measured by the priming task, would be 10. Following traditional stereotyping models that disregard directionality, the researcher concludes that the social group concept, A, brings B, C, and D to mind equally, because they possess equally strong associations with A, as measured by the bidirectional task. Our hypothetical researcher, then, designs a study in which participants respond to a person from social group A, with the prediction that attributes B, C, and D will have equally strong influences on the participant’s behavior. This prediction, however, would be incorrect. Following A’s presentation, D should not be activated at all, and C will be activated twice as strongly as B. Failing to consider directionality has led our researcher astray, leaving him or her puzzled, unclear about why the predictions were not borne out. It seems to us that careful consideration of directionality is absolutely necessary for understanding the validity of one’s design, measures, findings, and conclusions.

### Some Uses of the Publicly Available Data Matrices

The present work yielded several rich, versatile datasets exploring these stereotype networks. They can be used to create visual maps of the stereotype networks (as we did in this article’s striking image, using [68]). In addition to exploring further intricacies of stereotype directionality, these data matrices can be used to assess many other research questions. Using our raw data files, for example, one could assess whether our stimuli brought to mind other concepts we did not include in our coding. By looking at the *order* of generated responses, one could also test more fine-grained hypotheses about spreading activation—perhaps, for example, participants in the *gay* condition only generate “fashionable” as a response *after* they generate “feminine,” implying that the path of activation may be *gay* → *feminine* → *fashionable*. One could also assess test similar conceptual mediation pathways across conditions—knowing, for instance, how often participants in the *Black* condition responded with “poor” and how often participants in the *poor* condition responded with “criminal” gives one some indication of how *Black* may be indirectly connected to *criminal* through *poor*. Cluster and factor analyses of our data could reveal sets of attributes that tend to cluster within the stereotype network of a given group, giving one insights into different subtypes or partitions in the image of that group. Last but not least, our data matrices could be used to build actual connectionist neural networks for modeling many different stereotyping processes. We hope that these datasets will prove to be useful resources for future research.

### Our Connectionist-Informed Approach

More precise commitments and attention to theoretical models of stereotypes and stereotyping will fuel progress on many core issues of interest, including how stereotypes are activated, how they function, how they can be changed, and how they relate to prejudice and discrimination [7]. Although social psychologists have given limited attention to connectionism [10], adopting connectionism as a theoretical foundation in the present work led to novel and important insights. The lack of attention to connectionism within social psychology likely arises from mistakenly conflating common connectionist *methods* with connectionist *theories* [10, 19]. We surmise that the computer simulation methods often used by connectionists seem distant from the social behavior typically of interest to social psychologists. Computer models, however, are merely a method useful for testing connectionist theories of cognition [10, 19]. In our view, it is not the methods of connectionism that must transfer to social psychological science—it is the theoretical principles underlying connectionist approaches [15].

## Conclusion

Just as an engineer must learn about the underlying architecture of a bridge if he or she wants to understand how it was built, how it works, and how to tear it down, so must a psychologist learn about the underlying architecture of a stereotype if he or she wants to understand how it was learned, how it is activated, and how to change it. Adopting a theory-driven approach to understanding stereotype structure led us to predict and subsequently find evidence for heterogeneous stereotype directionality. We believe that consideration of stereotype directionality, and the cognitive mechanisms and structure of stereotypes more generally, will enable researchers and theorists to reap the benefits of greater theoretical clarity and make new scientific advancements and discoveries in the study of stereotypes, stereotyping, and other intergroup phenomena.

## Supporting Information

S1 File Study Materials. This contains sample experimental materials for Studies 1, 3, and 4.
(PDF)

S2 File Study 1 Raw Participant Data and Coding. This SPSS file contains the raw participant data from Study 1 and the coding for each participant’s responses.
(SAV)

S3 File All Data Matrices. This Excel file contains the compiled data matrices from Studies 1, 3, and 4, showing all connections among all the concepts within each study.
(XLSX)

S4 File Study 3 Raw Participant Data and Coding. This SPSS file contains the raw participant data from Study 3 and the coding for each participant’s responses.
(SAV)

S5 File Study 4 Raw Participant Data—Person Conditions. This Excel file contains the raw participant data from the “Person” conditions of Study 4.
(XLSX)

S6 File Study 4 Raw Participant Data—Woman Conditions. This Excel file contains the raw participant data from the “Woman” conditions of Study 4.
(XLSX)

## References

[1] Allport GW. The nature of prejudice. Reading, MA: Addison-Wesley; 1954

[2] Cox WTL, Abramson LY, Devine PG, Hollon SD. Stereotypes, prejudice, and depression: The integrated perspective. Perspect Psychol Sci, 2012;7: 427–449. 26168502 10.1177/1745691612455204

[3] Devine PG. Stereotypes and prejudice: Their automatic and controlled components. J Pers Soc Psychol, 1989;56: 5–18.

[4] Fiske ST. Stereotyping, prejudice, and discrimination In: Gilbert T, and Fiske ST, editors. Handbook of Social Psychology 4th ed, Boston, MA: McGraw-Hill 1998;2: 357–411.

[5] Pratto F, Sidanius J, Stallworth LM, Malle BF. Social dominance orientation: A personality variable predicting social and political attitudes. J Pers Soc Psychol, 1994;67: 741–763.

[6] Sue DW, Capodilupo CM, Torino GC, Bucceri JM, Holder AMB, Nadal KL, et al Racial microaggressions in everyday life: Implications for clinical practice. Am Psychol, 2007;62: 271–286. 17516773 10.1037/0003-066X.62.4.271

[7] Hilton JL, von Hippel W. Stereotypes. Annu Rev Psychol, 1996;47: 237–271. 15012482 10.1146/annurev.psych.47.1.237

[8] Hamilton DL, Sherman JW. Stereotypes In RS Wyer, TK Srull, (Eds.) Handbook of social cognition. Hillsdale, NJ England: Lawrence Erlbaum Associates, Inc 1994: 1–68.

[9] Smith ER. What do connectionism and social psychology offer each other? J Pers Soc Psychol, 1996;70: 893–912. 8656338 10.1037//0022-3514.70.5.893

[10] Smith ER. Distributed connectionist models in social psychology. Soc Personal Psychol Compass, 2009;3: 64–76.

[11] McClelland JL, Rogers TT. The parallel distributed processing approach to semantic cognition. Nat Rev Neurosci, 2003;4: 310–322. 12671647 10.1038/nrn1076

[12] McClelland JL, Botvinick MM, Noelle DC, Plaut DC, Rogers TT, Seidenberg MS, et al Letting structure emerge: connectionist and dynamical systems approaches to cognition. Trends Cogn Sci, 2010;14: 348–356. 10.1016/j.tics.2010.06.002 20598626 PMC3056446

[13] Rogers TT, McClelland JL. Parallel distributed processing at 25: Further explorations in the microstructure of cognition. Cogn Sci, 2014;38: 1024–1077. 10.1111/cogs.12148 25087578

[14] Freeman JB, Ambady N. A dynamic interactive theory of person construal. Psychol Rev, 2011;118. 10.1037/a0022327 21355661

[15] Garcia-Marques L, Santos AC, Mackie DM. Stereotypes: Static abstractions or dynamic knowledge structures? J Pers Soc Psychol, 2006;91: 814–831. 17059303 10.1037/0022-3514.91.5.814

[16] Smith ER, DeCoster J. Knowledge acquisition, accessibility, and use in person perception and stereotyping: Simulation with a recurrent connectionist network. J Pers Soc Psychol, 1998;74: 21–35. 9457773 10.1037//0022-3514.74.1.21

[17] Van Overwalle F. Social Connectionism. New York, NY: Psychology Press 2007.

[18] Van Rooy D, Van Overwalle F, Vanhoomissen T, Labiouse C, French R. A recurrent connectionist model of group biases. Psychol Rev, 2003;110: 536–563. 12885114 10.1037/0033-295x.110.3.536

[19] Seidenberg MS. Connectionist models and cognitive theory. Psychol Sci, 1993;4: 228–235.

[20] Mandelbrot BB. The fractal geometry of nature. Vol. 173 Macmillan 1983.

[21] Mac Cormac E, Stamenov MI, Fractals of brain, fractals of mind: in search of a symmetry bond. Vol. 7 John Benjamins Publishing, 1996.

[22] Katz D, Braly K. Racial stereotypes of one hundred college students. J Abnorm Soc Psychol, 1993;28: 280–290.

[23] Brewer M. A dual-process model of impression formation In: Srull T & Wyer R, editors. Advances in social cognition. Hillsdale N.J.: Erlbaum Associates 1988: 1–36.

[24] Fiske ST, Neuberg SL. A continuum of impression formation, from category-based to individuating processes: Influences of information and motivation on attention and interpretation In: Zanna MP, editor. Advances in Experimental Social Psychology, Volume 23 San Diego, CA: Academic Press 1990: 1–74.

[25] Blair IV, Judd CM, Sadler MS, Jenkins C. The role of Afrocentric features in person perception: Judging by features and categories. J Pers Soc Psychol, 2002;83: 5–25. 12088132

[26] Bodenhausen GV, Macrae CN. Stereotype activation and inhibition In: Wyer RS & Wyer RS, editors. Stereotype activation and inhibition: Advances in social cognition. Vol. 11 Psychology Press, 2013.

[27] Cox WTL, Devine PG. Stereotyping to infer group membership creates plausible deniability for prejudice-based aggression. Psychol Sci, 2014;25: 340–348. 10.1177/0956797613501171 24335602

[28] Cox WTL, Devine PG, Bischmann AA, Hyde JS. Inferences about sexual orientation: The role of stereotypes, faces, and the gaydar myth. J Sex Res, 2015: in press. 10.1080/00224499.2015.1015714 PMC4731319 26219212

[29] Eberhardt JL, Goff PA, Purdie-Vaughns VJ, Davies PG. Seeing black: Race, crime, and visual processing. J Pers Soc Psychol, 2004;87: 876–893. 15598112 10.1037/0022-3514.87.6.876

[30] Kawakami K, Dovidio J, Moll J, Hermsen S, Russin A. Just say no (to stereotyping): Effects of training in the negation of stereotypic associations on stereotype activation. J Pers Soc Psychol, 2000;78: 871–888. 10821195 10.1037//0022-3514.78.5.871

[31] Dotsch R, Wigboldus DHJ, van Knippenberg A. Biased allocation of faces to social categories. J Pers Soc Psychol, 2011;100: 999–1014. 10.1037/a0023026 21443368

[32] Hugenberg K, Bodenhausen GV. Ambiguity in social categorization: the role of prejudice and facial affect in race categorization. Psychol Sci, 2004;15: 342–345. 15102145 10.1111/j.0956-7976.2004.00680.x

[33] Johnson KL, Gill S, Reichman V, Tassinary LG. Swagger, sway, and sexuality: Judging sexual orientation from body motion and morphology. J Pers Soc Psychol, 2007;93: 321–334. 17723051 10.1037/0022-3514.93.3.321

[34] Hebb DO. The organization of behavior: A neuropsychological approach. New York: Wiley 1949.

[35] Tversky A. Features of similarity. Psychol Rev, 1977;84: 327–352.

[36] Cobos PL, López FJ, Caño A, Almaraz J, Shanks DR. Mechanisms of predictive and diagnostic causal induction. J Exp Psychol Anim Behav Process, 2002;28: 331–346. 12395491

[37] Fernbach PM, Darlow A, Sloman SA. Asymmetries in Predictive and Diagnostic Reasoning. J Exp Psychol Gen, 2011;140: 168–185. 10.1037/a0022100 21219081

[38] Waldmann MR, Holyoak KJ. Predictive and Diagnostic Learning Within Causal Models: Asymmetries in Cue Competition. J Exp Psychol Gen, 1992;121: 222–236. 1534834 10.1037//0096-3445.121.2.222

[39] Graves WW, Binder JR, Seidenberg MS. Noun-noun combination: Meaningfulness ratings and lexical statistics for 2160 word pairs. Behav Res Methods, 2013;45: 463–469. 10.3758/s13428-012-0256-3 23055162 PMC3663253

[40] Seidenberg MS, MacDonald MC, Saffran JR. Does grammar start where statistics stop? Science, 2002;298: 553–554. 12386323 10.1126/science.1078094

[41] Lionello-DeNolf KM. The search for symmetry: 25 years in review. Learn Behav, 2009;37: 188–203. 10.3758/LB.37.2.188 19380896 PMC2685068

[42] Dentico D, Cheung BL, Chang JY, Guokas J, Boly M, Tononi G, et al Reversal of cortical information flow during visual imagery as compared to visual perception. Neuroimage; 2014. 10.1016/j.neuroimage.2014.05.081 PMC4310722 24910071

[43] Bi GQ, Poo MM. Synaptic modification by correlated activity: Hebb's postulate revisited. Annu Rev Neurosci, 2001;24: 139–166. 11283308 10.1146/annurev.neuro.24.1.139

[44] Hinton GE, Salakhutdinov RR. Reducing the dimensionality of data with neural networks. Science, 2006;313(5786): 504–507. 16873662 10.1126/science.1127647

[45] Movellan JR, McClelland JL. Learning continuous probability distributions with symmetric diffusion networks. Cogn Sci, 1993;17: 463–496.

[46] Cacioppo JT, von Hippel W, Ernst JM. Mapping cognitive structures and processes through verbal content: The thought-listing technique. J Consult Clin Psychol, 1997;65: 928–940. 9420354 10.1037//0022-006x.65.6.928

[47] Jack AI, Roepstorff A. Introspection and cognitive brain mapping: from stimulus—response to script—report. Trends Cogn Sci, 2002;6: 333–339. 12140083 10.1016/s1364-6613(02)01941-1

[48] McRae K, Cree GS, Seidenberg MS, McNorgan C. Semantic feature production norms for a large set of living and nonliving things. Behav Res Methods, 2005;37: 547–559. 16629288 10.3758/bf03192726

[49] Preece PE. Mapping cognitive structure: A comparison of methods. J Educ Psychol, 1976;68: 1–8.

[50] Matthews C, Hill C. Gay until proven straight: Perceptions of male interior designers from male practitioner and student perspectives. J Interior Design, 2011;36: 15–34.

[51] Shelp SG. Gaydar: Visual detection of sexual orientation among gay and straight men. J Homosex, 2002;44: 1–14. 12856753 10.1300/j082v44n01_01

[52] Gaertner SL, Dovidio JF. The aversive form of racism In: Dovidio JF & Gaertner SLm editors. Prejudice, discrimination, and racism. Orlando, FL: Academic Press 1986: 61–89.

[53] Devine PG, Elliot AJ. Are racial stereotypes really fading? The Princeton trilogy revisited. Pers Soc Psychol Bull, 1995;21: 1139–1150.

[54] Kite ME, Deaux K. Gender belief systems: Homosexuality and the implicit inversion theory. Psychol Women Q, 1987;11: 83–96.

[55] GraphPad QuickCalcs. Available: http://graphpad.com/quickcalcs/contingency1.cfm. Accessed 2014 Dec 13.

[56] Wilson DB. Practical meta-analysis effect size calculator. 2001. Available: http://www.campbellcollaboration.org/resources/effect_size_input.php. Accessed 2014 Dec 13.

[57] Lipsey MW, Wilson DB. Practical meta-analysis. Vol. 49 Thousand Oaks, CA: Sage publications, 2001.

[58] Borenstein M, Hedges LV, Higgins JPT, Rothstein HR. Introduction to Meta-Analysis. John Wiley & Sons, Ltd, Chichester, UK 2009.

[59] Russo V. The celluloid closet: Homosexuality in the movies. New York: Arbor House Publishing 1987.

[60] Johnson A. 'Coastie' song, video spark debate at UW. Journal Sentinel. 2009, 12 19.

[61] Devine PG, Sherman S. Intuitive Versus Rational Judgment and the Role of Stereotyping in the Human Condition: Kirk or Spock? Psychol Inq, 1992.

[62] Rothbart M, Taylor M. Category labels and social reality: Do we view social categories as natural kinds? In: Semin GR & Fiedler K, editors. Language, interaction and social cognition. Thousand Oaks: Sage Publications, Inc 1992: 11–36.

[63] Bodenhausen GV. Stereotypic biases in social decision making and memory: Testing process models of stereotype use. J Pers Soc Psychol, 1988;55: 726–737. 3210142 10.1037//0022-3514.55.5.726

[64] Cox WTL, Devine PG, Plant EA, Schwartz LL. Toward a comprehensive understanding of officers’ shooting decisions: No simple answers to this complex problem. Basic Appl Soc Psych, 2014;36: 356–364.

[65] Plant E, Peruche B, Butz DA. Eliminating automatic racial bias: Making race non-diagnostic for responses to criminal suspects. J Exp Soc Psychol, 2005;41: 141–156.

[66] Eagly AH, Karau SJ. Role congruity theory of prejudice toward female leaders. Psychol Rev, 2002;109: 573–598. 12088246 10.1037/0033-295x.109.3.573

[67] Bargh JA, Chartrand TL. The mind in the middle. Handbook of research methods in social and personality psychology, 2000: 253–285.

[68] Smith M, Milic-Frayling N, Shneiderman B, Mendes Rodrigues E, Leskovec J, Dunne C. NodeXL: a free and open network overview, discovery and exploration add-in for Excel 2007/2010. http://nodexl.codeplex.com/ from the Social Media Research Foundation. Available: http://www.smrfoundation.org. 2010. Accessed 2014 Dec 13.

