# Supplementary material for: Stereotypes Possess Heterogeneous Directionality: A Theoretical and Empirical Exploration of Stereotype Structure and Content
Source: PLoS One. 2015 Mar 26;10(3):e0122292. doi: 10.1371/journal.pone.0122292 (PMC4374885; doi:10.1371/journal.pone.0122292)
Supplement: S1 File — This contains sample experimental materials for Studies 1, 3, and 4. (PDF) [file pone.0122292.s001.pdf]

Study 1 Materials Sample

We want to know what comes to an individual's mind when they think of certain traits. Please give your **gut response** to the question below. Just list the first things that pop into your mind; do not censor them or worry about how they sound. There are no right or wrong answers. We are not interested in individual participants, we are only interested in the items in the survey and what is associated with them. Also, please remember all responses are **anonymous**. When you are done, we will separate this sheet from the questionnaire so it won't be tied to your name. It will never be associated with you in any way.

List the first five things that come to your mind when you picture:

A man who is gay.

1) ______________________________
2) ______________________________
3) ______________________________
4) ______________________________
5) ______________________________

Circle:
Your gender: M F
You have lived in the U.S.: More than 4 Years Fewer than 4 years

We want to know what comes to an individual's mind when they think of certain traits. Please give your **gut response** to the question below. Just list the first things that pop into your mind; do not censor them or worry about how they sound. There are no right or wrong answers. We are not interested in individual participants, we are only interested in the items in the survey and what is associated with them. Also, please remember all responses are **anonymous**. When you are done, we will separate this sheet from the questionnaire so it won't be tied to your name. It will never be associated with you in any way.

List the first five things that come to your mind when you picture:

A man who is Black.

1) ______________________________
2) ______________________________
3) ______________________________
4) ______________________________
5) ______________________________

Circle:
Your gender: M F
You have lived in the U.S.: More than 4 Years Fewer than 4 years

Study 3 Materials Sample

We want to know what comes to an individual’s mind when they think of certain traits. Please give your **gut response** to the question below. Just list the first things that pop into your mind; do not censor them or worry about how they sound. There are no right or wrong answers. We are not interested in individual participants, we are only interested in the items in the survey and what is associated with them. Also, please remember all responses are **anonymous**. When you are done, we will separate this sheet from the questionnaire so it won't be tied to your name. It will never be associated with you in any way.

List the first five things that come to your mind when you picture:

A man who is athletic.

1) ______________________________
2) ______________________________
3) ______________________________
4) ______________________________
5) ______________________________

Circle:
Your gender: M F Does the above sentence describe you? Y N
You have lived in the U.S.: More than 4 Years Fewer than 4 years

We want to know what comes to an individual’s mind when they think of certain traits. Please give your **gut response** to the question below. Just list the first things that pop into your mind; do not censor them or worry about how they sound. There are no right or wrong answers. We are not interested in individual participants, we are only interested in the items in the survey and what is associated with them. Also, please remember all responses are **anonymous**. When you are done, we will separate this sheet from the questionnaire so it won't be tied to your name. It will never be associated with you in any way.

List the first five things that come to your mind when you picture:

A man who is poor.

1) ______________________________
2) ______________________________
3) ______________________________
4) ______________________________
5) ______________________________

Circle:
Your gender: M F Does the above sentence describe you? Y N
You have lived in the U.S.: More than 4 Years Fewer than 4 years

Study 4 Materials Sample

We want to know what comes to an individual's mind when they think of certain traits. Please give your **gut response** to the question below. Just list the first things that pop into your mind; do not censor them or worry about how they sound. There are no right or wrong answers. We are not interested in individual participants, we are only interested in the items in the survey and what is associated with them. Also, please remember all responses are **anonymous**. When you are done, we will separate this sheet from the questionnaire so it won't be tied to your name. It will never be associated with you in any way.

List the first five things that come to your mind when you picture:

A person who drinks alcohol.

1) ______________________________
2) ______________________________
3) ______________________________
4) ______________________________
5) ______________________________

Circle:
Your gender: M F Does the above sentence describe you? Y N
You have lived in the U.S.: More than 4 Years Fewer than 4 years

We want to know what comes to an individual's mind when they think of certain traits. Please give your **gut response** to the question below. Just list the first things that pop into your mind; do not censor them or worry about how they sound. There are no right or wrong answers. We are not interested in individual participants, we are only interested in the items in the survey and what is associated with them. Also, please remember all responses are **anonymous**. When you are done, we will separate this sheet from the questionnaire so it won't be tied to your name. It will never be associated with you in any way.

List the first five things that come to your mind when you picture:

A person who has a beard.

1) ______________________________
2) ______________________________
3) ______________________________
4) ______________________________
5) ______________________________

Circle:
Your gender: M F Does the above sentence describe you? Y N
You have lived in the U.S.: More than 4 Years Fewer than 4 years
